# Low Levels of Procalcitonin Are Related to Decreased Antibiotic Use in Children Hospitalized Due to Influenza

**DOI:** 10.3390/diagnostics12051148

**Published:** 2022-05-05

**Authors:** August Wrotek, Oliwia Wrotek, Teresa Jackowska

**Affiliations:** 1 Department of Pediatrics, Centre of Postgraduate Medical Education, Marymoncka 99/103, 01-813 Warsaw, Poland; 2 Department of Pediatrics, Bielanski Hospital, Cegłowska 80, 01-809 Warsaw, Poland; 3 Student Research Group at the Bielanski Hospital, 01-809 Warsaw, Poland; oliwia.wrotek@gmail.com

**Keywords:** procalcitonin, C-reactive protein, paediatric, influenza, pneumonia, sensitivity and specificity

## Abstract

Procalcitonin increases in bacterial infections, which are often suspected (though rarely confirmed) in paediatric influenza. We retrospectively verified procalcitonin’s usefulness in antibiotic guidance in children hospitalized due to laboratory-confirmed influenza. The ROC curve analysis evaluated procalcitonin’s performance in terms of antibiotic implementation or continuation in patients who were naive or had been receiving antibiotic treatment prior to hospital admission. We also assessed the procalcitonin’s usefulness to predict lower-respiratory-tract infections (LRTI), the presence of radiologically confirmed pneumonia, an intensive care unit transfer and a fatal outcome. Multiple regression models were built to verify the previously reported procalcitonin cut-off values. The study enrolled 371 children (median age 33 months). The AUC (area under the curve) for antibiotic implementation reached 0.66 (95%CI: 0.58–0.73) and 0.713 (95%CI: 0.6–0.83) for antibiotic continuation; optimal cut-offs (0.4 and 0.23 ng/mL, respectively) resulted in a negative predictive value (NPV) of 79.7% (95%CI: 76.2–82.9%) and 54.6% (95%CI: 45.8–63%), respectively. The use of 0.25 ng/mL as a reference decreased the odds of antibiotic treatment by 67% (95%CI: 43–81%) and 91% (95%CI: 56–98%), respectively. Procalcitonin showed lower AUC for the prediction of LRTI and pneumonia (0.6, 95%CI: 0.53–0.66, and 0.63, 95%CI: 0.56–0.7, respectively), with a moderately high NPV in the latter case (83%, 95%CI: 79.3–86.1%). Procalcitonin use may decrease the antibiotic frequency in hospitalized influenza cases both in terms of antibiotic administration and continuation. Procalcitonin concentrations may suggest bacterial suprainfections at lower concentrations than in adults, and a focus on its rule-out value is of special interest.

## 1. Introduction

Influenza is highly contagious disease, and the estimates of influenza attack rate in children vary between 8.7% [1] and 15.2% [2]. Its course may be benign to severe, from self-limiting to complicated; it may possibly lead to hospitalization or even death [3]. Global assessments report around 109.5 million cases in children under 5 years of age annually, with 870,000 acute lower-respiratory-tract infection (ALRI) hospitalizations, and 15,300 in-hospital deaths [4]. Complications are seen frequently, especially in hospitalized children, in whom pneumonia and acute otitis media may affect up to 10–12% and 22–30% of patients, respectively [5]. Although influenza is a viral infection, patients also run a high risk of bacterial coinfections, which are often responsible for complications and can, thus, become life-threatening conditions [6].

The true frequency of bacterial coinfections remains unknown; a confirmation of bacterial presence (with a culture or a molecular assay) in a sample from a sterile site lacks sensitivity, while other diagnostic methods (e.g., antigen tests) lack specificity [7]. The use of antibiotics is high, especially in hospitalized cases (79% in the general adult population, and 97% in the cases of pneumonia), but only few cases of bacterial infection are confirmed [8]. A global assessment, which included over 6000 children hospitalized during the A/H1N1 pandemic, reported the use of antibiotics in 45.2% of patients [9]. Local data report higher percentages, which can reach (in Korea, for example) 61% in children hospitalized due to influenza without pneumonia, and 94% in influenza cases complicated with pneumonia [10]. The use of antibiotics, despite its obvious benefits, carries the risk of adverse events, and various educational programs are being implemented in order to decrease the overuse of antimicrobials [11]. Thus, clinically relevant biomarkers are imperative for antibiotic stewardship.

Procalcitonin (PCT) is a precursor of calcitonin, and its concentrations increase mainly in bacterial infections, when cytokines mediate an inflammatory response [12,13]. At the moment, PCT is one of the most widely investigated and used inflammatory markers with the scope of distinguishing bacterial from viral infections, including in pneumonia and other lower-respiratory-tract infections (LRTI) [14,15]. Procalcitonin levels have also been investigated in influenza patients, although the majority of the studies focused on establishing the procalcitonin cut-off values that would confirm bacterial infections [16,17,18,19,20,21], and some were conducted in intensive care unit (ICU) settings [20,21]. However, the vast majority of paediatric patients with influenza are hospitalized at general paediatric wards [9], where the number of confirmed bacterial coinfections is much lower than in the ICU, yet the need for the use of antibacterials remains relatively high [9]. An inverse approach towards inflammatory markers was applied in certain other studies on paediatric pneumonia or influenza-related pneumonia and focused on the negative predictive value of PCT. It seems justified in paediatric influenza as well [22,23]; since a confirmation of bacterial suprainfection is rather scarce, on the other hand, an attempt to establish rule-out PCT values is an achievable goal. A lack of confirmation of bacterial infection does not always mean that antibiotic therapy is not necessary, while a cure without antibiotics may be treated as a lack of or just a minor coinfection (i.e., not requiring antibacterials) and represents a practical approach. Well-established procalcitonin cut-off values are crucial for antibiotic stewardship, but a broad database on PCT’s levels with its clinical significance needs to be established prior to the implementation of procalcitonin-based protocols in a clinical practice. In this study, we sought to answer the question of the usefulness of procalcitonin in excluding the need for antibiotic treatment in children with laboratory-confirmed influenza (LCI).

## 2. Materials and Methods

### 2.1. Study Design

The study had a retrospective design and included patients hospitalized at the Department of Paediatrics, Bielanski Hospital, Warsaw, Poland, in the period between January 2013 and December 2019 (7 consecutive years). Due to its retrospective character, the patients’, as well as the parents’/tutors’, consent was waived.

### 2.2. Data Retrieval

Electronical medical charts of hospitalized patients were retrospectively reviewed, and patients diagnosed with influenza were eligible for the study. The inclusion criteria consisted of the ICD-10 (the International Classification of Diseases, 10th Revision) codes of J10 (+extensions)—influenza due to an identified virus, and in the case of all patients diagnosed with ICD-10 code J11 (+extensions), influenza due to an unidentified virus. The medical charts were revised in search of an existing influenza confirmation, as a bias related to the ICD coding might have taken place.

### 2.3. Inclusion/Exclusion Criteria

We enrolled only laboratory-confirmed cases of influenza, and the presence of an aetiological factor was proved either with the use of a rapid influenza diagnostic test (RIDT) or a reverse transcription–polymerase chain reaction (RT-PCR), with the latter being the reference method. Both RIDT and RT-PCR were performed on nasopharyngeal swabs taken immediately at hospital admission; in order to exclude a nosocomial origin of the infection, a time limit for the first signs/symptoms’ presentation was set as within the first 48 hours after admission. We did not include children younger than 30 days. Patients were excluded if they had immune deficiency (hereditary, acquired or resulting from an immunosuppressive treatment), diabetes, proliferative disease or a history of a proliferative disease, cystic fibrosis, haemodynamically significant heart disease, burn or a history of a recent burn (up to 7 days prior to hospitalization); the exclusion criteria also contained a lack of full knowledge on the disease course (e.g., discharge on parental request).

### 2.4. Patients’ Characteristics

Demographical, laboratory and clinical characteristics were collected, including age and gender; duration of signs/symptoms prior to hospitalization, duration of fever prior to hospitalization and antibacterial therapy prior to hospitalization; breath rate and heart rate at hospital admission; C-reactive protein (CRP), procalcitonin, white blood cell count (WBC) and absolute neutrophil count (ANC) at admission and blood cultures taken (and the results, including antibiogram); length of hospital stay (LOS), data on rehospitalization or a consultation in the emergency department of the Bielanski Hospital within a month after the discharge (including the treatment performed), need for intensive care unit (ICU) transfer and a fatal outcome.

### 2.5. Laboratory Procedures

Blood analysis was performed immediately after hospital admission, and only patients who had their procalcitonin level measured were included in the study. Procalcitonin concentrations were measured with the use of the Cobas e411 (until 4 August 2016), and the Cobas 6000 (from 5 August 2016) analysers (Roche Diagnostics Ltd., Switzerland). All procedures were performed according to the manufacturer’s instructions.

### 2.6. Study Groups

Based on a history of their recent antibacterial therapy, patients were assigned into two groups: children who had not received antibacterial therapy within 14 days prior to hospital admission created the No-PAT (no prior antibiotic therapy) group, while children who had had a recent history or were on ongoing antibacterial therapy were assigned to the PAT (prior antibiotic therapy) group.

### 2.7. Study Endpoints

The study endpoints included the following: 1. the use of antibacterials, 2. positive blood culture, 3. ICU transfer, 4. fatal outcome, 5. lower-respiratory-tract involvement and 6. radiologically confirmed pneumonia.

In the No-PAT group, the patients were divided into two subgroups based on the treatment they received during the hospitalization (antibiotic versus no antibiotic group). In the PAT group, we compared patients who had antibiotic continued (or another antibiotic was implemented) against those who had the antibiotic discontinued.

### 2.8. Treatment Success

a. If a child did not receive antibiotics during the hospitalization, an improvement was observed, the patient was discharged and was not readmitted within a month due to influenza complications that would require antibiotic treatment, the treatment was considered successful, and the diagnosis of a sole influenza was a correct one. In theory, although with low probability, a minor bacterial coinfection without clinical effects could have taken place.

b. If a patient received antibiotics during the hospitalization, irrespectively of the indications (the decision of the antibiotic implementation was made on physician’s discretion only and based in each case upon the clinical picture), the benefits of antibacterial therapy could not be excluded. No analysis on particular indications for antibiotic treatment was performed.

### 2.9. Treatment Failure

All children who had not received antibiotics during hospitalization but were later rehospitalized or consulted at the emergency department of the Bielanski Hospital, at which time an antibiotic treatment was implemented, were considered to have received unsuccessful treatment and were assigned to the group who might have benefited from antibiotic therapy during the initial hospitalization.

With regard to the rest of the endpoints (i.e., positive blood culture, ICU transfer, fatal outcome, lower-respiratory-tract involvement, radiologically confirmed pneumonia), the analysis was performed in the whole study group, and no protective effect of prior antibiotic therapy was assumed. Lower-respiratory-tract involvement was defined upon a final diagnosis according to the ICD-10 codes: J10.0 for influenza and pneumonia, J12 to J18 (+extensions) for pneumonia, J20 (+extensions) for bronchitis, J21 (+extensions) for bronchiolitis and J22 for unspecified lower-respiratory-tract infection; the diagnosis was made by a treating physician.

Radiologically confirmed pneumonia was defined as the presence of radiological abnormalities consistent with pneumonia (dense or fluffy opacities, minor infiltrates, linear and patchy densities).

### 2.10. Statistical Analysis

The Kolmogorov–Smirnov test was employed to assess the data distribution, and continuous data were presented as a mean and a standard deviation or a median and an interquartile range, according to the distribution. A corresponding parametric (Student *t* test) or non-parametric test (Mann–Whitney *U* test) was used to compare the groups, and Fisher’s exact test was used for frequency comparison. A receiver-operating curve (ROC) analysis was performed for calculations of the optimal procalcitonin cut-off value, for which sensitivity, specificity and positive and negative predictive value (PPV and NPV, respectively) were established. A multiple regression model was created in order to estimate the odds ratios (ORs) with 95% confidence intervals (95%CI) for categorical variables. The LOS was transformed from a continuous into a categorical variable upon the median value. A decrease in antibiotic treatment odds in children with procalcitonin lower than the cut-off values was calculated by subtracting 1 from the odds ratio of receiving antibiotic (No-PAT group) or from the odds ratio of antibiotic continuation (PAT group). Since different optimal cut-off values might have been expected in the ROC analysis, in order to facilitate comparisons, we verified the decrease in the odds of antibiotic treatment only for the previously assumed (based on the literature search) cut-off values of 0.25, 0.5 and 1 ng/mL. The *p* values under 0.05 were considered to present a statistical significance. The analysis was performed with Statistica 13.1 software (Statsoft, Tulsa, OK, USA). Confidence intervals for sensitivity, specificity, PPV and NPV were calculated with the use of a diagnostic test evaluation calculator available at https://www.medcalc.org/calc/diagnostic_test.php (10 February 2021).

### 2.11. Ethics Committee

The research was granted permission by the Ethic Committee at the Centre of Postgraduate Medical Education in Warsaw (permission number 13/2021, issued on 10 March 2021).

## 3. Results

**Figure 1:**
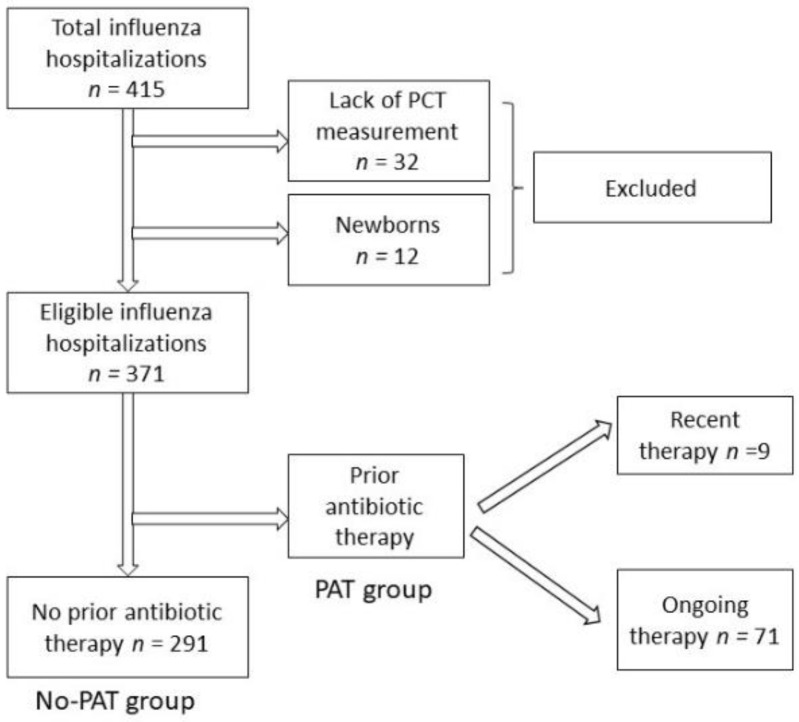
A flow chart of the patients included in the study. Abbreviations: PCT—procalcitonin, PAT group—prior antibiotic therapy, No-PAT—no prior antibiotic therapy. Influenza type A was diagnosed in 268 (72.2%) cases, type B in 90 (24.3%), while type A and B coinfection occurred in 13 (3.5%) cases.

In total, 415 children were hospitalized due to LCI in the period of 2013–2019, and PCT levels were measured in 383 (92.3%) children, including 12 newborns. The final study group consisted of 371 patients, including 291 patients (median age 33 months) without previous antibacterial therapy (No-PAT group) and 80 children (median age 34 months) on an ongoing (71 patients) antibiotic therapy or with a recent history (9 patients) of antibiotic therapy (PAT group) (Figure 1). There were no readmissions or emergency department consultations after hospital discharge that would result in antibiotic implementation.

Blood cultures were taken in 320 patients (86.3%) and showed a bacterial growth in seven cases (2.2%), with three cases of *Staphylococcus hominis*, and single cases of *Staphylococcus epidermidis*, *Streptococcus mitis/oralis*, *Micrococcus luteus*, and *Micrococcus* sp. A final diagnosis of a generalized bacterial infection was made only in one patient (where *St. hominis* was found in the blood), while the other cases were verified and identifies as a sample contamination, including two cases (*St. hominis* and *Micrococcus* sp.) where a generalized infection was judged to be extremely unlikely, and antibiotics were not started after the initial alert information on a positive blood culture from the microbiological laboratory. One patient (0.3%) required an ICU transfer because of respiratory failure. There were no fatal cases.

Children with prior antibacterial therapy (PAT group) had been presenting signs/symptoms for a longer period of time (median 4.5 vs. 2 days, *p* < 0.01), including a longer period of fever (median 4 vs. 2 days, *p* < 0.01), and required longer LOS (6 versus 5 days, *p* = 0.013). Antibiotics during hospitalization were administered more frequently in the PAT group (61%, 49 out of 80) than in the No-PAT group (29%, 84 out of 291), and the difference was statistically significant (Fisher’s exact test *p* < 0.05). In the whole study group, the antibiotic therapy frequency reached 35.9% (133 out of 371). No other significant differences between the PAT and the No-PAT groups, including the PCT concentrations, were observed (Table 1).

In the No-PAT group, children who received antibiotic treatment were younger (17.5 vs. 39 months, *p* < 0.01) and had a higher breath rate and heart rate at admission (29 vs. 24 per minute, *p* < 0.01, and 120 vs. 108 per minute, *p* < 0.01, respectively), but did not differ with regards to the duration of signs/symptoms or fever. They also required longer LOS (7 vs. 5 days, *p* < 0.01) and had higher PCT levels (0.3 vs. 0.17 ng/mL, *p* < 0.01), as well as higher CRP (10.77 vs. 4.21 mg/L, *p* < 0.01) and WBC (9.27 vs. 7.26*10^3/µL, *p* < 0.01) (Table 2).

**Figure 2:**
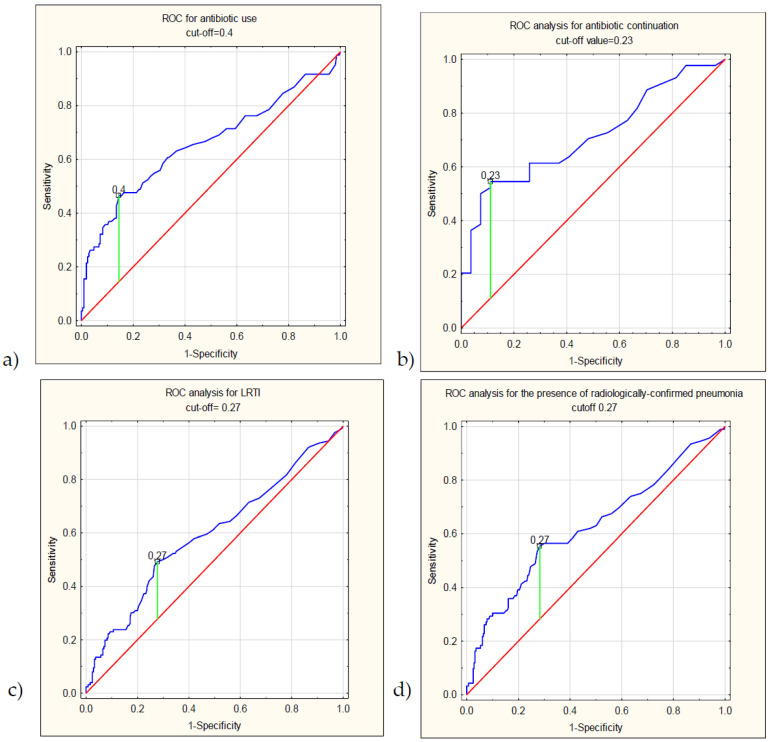
The results of the ROC-curve analysis, graphs showing the ROC analysis for (**a**) antibiotic implementation (No-PAT group), (**b**) antibiotic continuation (PAT group), (**c**) lower-respiratory-tract infection and (**d**) presence of radiologically confirmed pneumonia.

The ROC curve analysis for the prediction of the use of antibacterials (Figure 2) in the No-PAT group showed an AUC of 0.658 (95%CI: 0.581 to 0.734, *p* < 0.01) with a negative predictive value of NPV = 79.73% (95%CI: 76.18% to 82.87%). An optimal cut-off was calculated at 0.4 ng/mL and showed a sensitivity of 46.43% (95%CI: 35.47% to 57.65%), and a specificity of 85.51% (95%CI: 79.96% to 90.00%), with a PPV = 56.52% (95%CI: 46.49% to 66.04%). At the three other cut-offs reported most commonly in other studies on PCT (0.25, 0.5 and 1 ng/mL), the NPV reached 80.23% (95%CI: 75.61% to 84.15%), 77.54% (95%CI: 74.43% to 80.38%) and 76.15% (95%CI: 73.70% to 78.45%), respectively (Table 3).

In children who had previously received antibiotics (PAT group), the AUC for antibiotic continuation was 0.713 (95%CI: 0.6–0.83, *p* < 0.01), with a sensitivity of 54.55% (95%CI: 38.85% to 69.61%), specificity of 88.89% (95%CI: 70.84% to 97.65%), PPV 88.89% (95%CI: 72.69% to 96.01%) and an NPV of 54.55% (95%CI: 45.82% to 63.00%) at the optimal cut-off value established at 0.23 ng/mL. The NPV reached 53.33% (95%CI: 44.94% to 61.55%), 44.83% (95%CI: 40.06% to 49.70%) and 42.86% (95%CI: 39.48% to 46.30%) at 0.25, 0.5 and 1 ng/mL, respectively.

The PCT remained insignificant in terms of a positive blood culture or an ICU transfer.

In the multiple regression models constructed for the No-PAT and the PAT groups separately, lower PCT levels were related to lower odds of an antibiotic treatment in both groups. In children without previous antibacterial therapy, the odds ratio of receiving antibiotic treatment was decreased by 67% (OR = 0.33, 95%CI: 0.19 to 0.57, *p* < 0.01) in those who had PCT lower than 0.25 ng/mL, while in children with an ongoing antibiotic therapy (at hospital admission), lower PCT values were related to a 91% decrease in the odds of antibiotic continuation (OR = 0.092, 95%CI: 0.02 to 0.44, *p* < 0.01). A verification of other PCT cut-off values revealed a 78% and 88% decrease in the odds of antibiotic therapy in the No-PAT group (at PCT 0.5 and 1 ng/mL, respectively). In the PAT group, there was an 88% decrease in the odds of antibiotic treatment at 0.5 ng/mL, while all patients with PCT 1 ng/mL received antibacterial treatment (Table 4).

The area under the curve (AUC) for the prediction of LRTI was 0.6 (95%CI: 0.53–0.66, *p* < 0.001), and the cut-off concentration 0.27 ng/mL had a sensitivity of 49.21% (95%CI: 40.19% to 58.26%), a specificity of 72.24% (95%CI: 66.19% to 77.76%), PPV 47.69% (95%CI: 41.07% to 54.40%) and the NPV of 73.44% (95%CI: 69.61% to 76.96%). For radiologically confirmed pneumonia, the AUC was 0.626 (95%CI: 0.56–0.7, *p* < 0.01), and a cut-off 0.27 ng/mL showed a sensitivity of 55.43% (95%CI: 44.70% to 65.81%), a specificity of 71.68% (95%CI: 66.01% to 76.89%), PPV 39.23% (95%CI: 33.20% to 45.61%) and NPV 82.99% (95%CI: 79.33% to 86.11%) (Table 3).

## 4. Discussion

Our study shows that procalcitonin might be helpful in guiding antibiotic therapy in children with influenza, and special interest should be on the rule-out performance of procalcitonin levels. Attention might be focused on PCT as a predictor both of antibiotic implementation and of antibiotic withdrawal in children who were previously receiving antibacterial therapy.

The negative predictive value (NPV) of PCT regarding antibiotic implementation that was observed in our study is promising and reveals comparable, although slightly lower values than those reported in the previous studies (here, 0.8 at cut-off of 0.4 ng/mL, 0.8 and 0.78 at 0.25 ng/mL, and 0.5 ng/mL, respectively). The study by Li and colleagues included 3180 children under five years of age, and PCT showed an NPV of 0.852 (at a cut-off value of 0.52 ng/mL) regarding a proven bacterial coinfection [16]. Li et al. calculated the AUC of PCT for the prediction of a bacterial coinfection to be 0.801 [95%CI: 0.772–0.855], and only a CRP–PCT combination demonstrated a higher AUC 0.893 [95%CI: 0.852–0.934], but in turn, the CRP–PCT had a slightly lower NPV (0.846) [16]. Another large study, by Rodriguez et al., conducted at 148 ICUs in Spain, included 972 patients (>15 years of age), and revealed a higher NPV of PCT: 0.919 (at cut-off of 0.29 ng/mL) with an AUC = 0.716 [95%CI: 0.67–0.75] [17]. Other studies, referring only to adults, show NPVs from 0.84 (at a cut-off >1.5 ng/mL) with an AUC = 0.698 [95%CI: 0.523–0.873], in 60 emergency department patients reported by Ahn [18], to 1.0 in 38 adult pneumonia patients presented by Guervilly (at a cut-off of 0.5 ng/mL) [19]. Cucqumelle et al. conducted research at 23 French ICUs, enrolling 103 adults with AH1N1 pneumonia, complicated with a bacterial coinfection or not, and found an NPV = 0.91 (at a cut-off = 0.8 ng/mL) with an AUC = 0.90 (95%CI: 0.74–1) [20]. Similarly to Guervilly, 100% NPV (at a cut-off of 0.8 ng/mL) with an AUC = 0.88 [95%CI: 0.73–1.01] was observed by Ingram in 25 adults in the ICU, although the control group consisted of bacterial community-acquired pneumonia, not influenza pneumonia coinfected with bacteria [21]. Pfister et al. analysed 46 adults with pneumonia (26 with AH1N1) hospitalized in intensive care, alongside with the previously published data [20,21,24,25,26], thus creating a group of 161 patients [27]. Here, the isolated AH1N1 (*n* = 84) was compared against a bacterial pneumonia group (consisting of 37 patients with isolated bacterial pneumonia and 40 with mixed bacterial/influenza infection; no subgroup analysis was performed); after excluding hospital-acquired cases and immunodeficient patients, and the PCT’s NPV was 0.822 (at a cut-off of 0.5 ng/mL), which is closer to our outcomes.

The AUC observed in our trial was lower (here, AUC = 0.658) than the ones shown above; however, we can compare our results only to the studies that sought to confirm, not exclude, a bacterial infection. This may be related to the risk of unnecessary treatment in patients without a (proven) bacterial coinfection in our group of patients; on the other hand, a study protocol that focuses on confirmed bacterial infections only, may easily miss non-septic patients who need antibiotic treatment. Thus, we chose a negative predictive value model, following the one used by Stockmann and colleagues for antibiotic guidance in children with pneumonia, a disease that meets a similar problem with its aetiology confirmation [22]. In pneumonia, the aetiological confirmation is uncertain due to the very low sensitivity of blood culture and a risk of false positive results on one hand, while the available diagnostic methods do not allow the exclusion of a bacterial aetiology, on the other hand [28,29,30,31]. The study design proposed by Stockmann, based on a clinical observation of patients treated without antibiotics, allows to confirm not the lack of a bacterial infection itself but the lack of a necessity for antibacterial treatment—a finding that is highly desired in the field of paediatrics [22]. Similar obstacles with regards to bacterial infections are met in the case of influenza, the risk of a bacterial coinfection is high and antibiotics are used very often, but confirmed bacterial coinfections are seen less frequently [32].

In the aforementioned study by Li, a bacterial coinfection was found in 7.1% of hospitalized cases [16], while a systematic review shows differences varying between 2% in newborns [32,33] and 65% in adults [32,34], although no age relationship can be drawn. In our group of patients, we confirmed only one case of a bacterial coinfection, although blood cultures were taken in the vast majority of patients (over 86%) and revealed bacterial growth in seven cases (2.2%). Moreover, the hypothesized suprainfection was caused by bacteria that are not typical for coinfections, while other bacterial growths were finally verified as sample contaminations, which clearly reflects the obstacles in everyday clinical practice met by any attempts to confirm a bacterial suprainfection. The use of antibiotics was moderate: 35.9% of patients obtained antibacterial treatment, which is lower than the global frequency during the 2009 pandemic reported by Muthuri (45%) [9]. Although the antibiotic use in our study seems to be reasonably low, it highlights the need for antibacterials in a significant percentage of paediatric patients with influenza, and a wide use of clinical tools such as PCT guidance might further decrease the frequency of antibiotic therapy.

It needs to be emphasized that there are many potential factors affecting comparisons between the studies, including various study settings or age groups. Nevertheless, procalcitonin levels differ between the coinfected and sole influenza cases. The study by Li et al. showed that PCT was significantly higher in children with a proven bacterial coinfection (1.46 versus 0.21 ng/mL) [16], while the difference demonstrated in our study is less striking (0.3 vs. 0.17 ng/mL, *p* < 0.01). Adult studies revealed an even stronger diversity (Rodriguez: 2.4 vs. 0.5 ng/mL; Ahn: 3.45 vs. 0.15 ng/mL; Guervilly: 6.5 vs. 1 ng/mL; Cucqumelle: 29.5 vs. 0.5 ng/mL), but this might be attributable to the factors mentioned above, mainly concerning the group selection [17,18,19,20].

There are discrepancies regarding the cut-off values of PCT also. In our study, the cut-off for antibiotic implementation set at 0.4 ng/mL proved to best fit the model (according to the Youden index) and is lower than the cut-off in the study by Li (cut-off = 0.52 ng/mL), but different study protocols were used here. Higher cut-offs, which varied between 0.8 [21] to 1.5 ng/mL [18], may be related to, inter alia, the study setting and need to be considered with extreme precaution, since the lack of laboratory-confirmed bacterial infection does not exclude the need for antibiotic therapy. In children with a viral LRTI admitted to the intensive care unit, PCT = 1.4 ng/mL had an NPV = 0.76 for excluding a bacterial coinfection [35]. On the other hand, a prospective study by Esposito conducted in children with pneumonia used a cut-off value of 0.25 ng/mL as a reference value under which antibiotics do not need to be implemented [36]. Similar findings were presented by Stockmann et al. in another study on pneumonia that showed PCT’s negative predictive value of 0.96 [95%CI: 0.93–0.99] in differentiating typical bacterial pneumonia (in confirmed cases) from pneumonia of other aetiology [22]. Stockmann included 532 children hospitalized due to radiologically confirmed pneumonia, and interestingly, children with sole influenza pneumonia (*n* = 23) had a higher median value of PCT than did other viral pneumonia patients (PCT = 0.62, 95%CI: 0.13–2.51) [22]. Moreover, the type of the bacterial coinfection may influence the PCT levels, since patients with a LRTI due to Gram-negative bacteria tend to have higher PCT values than those infected with Gram-positive bacteria [37].

Obviously, PCT is not a golden standard for the purpose of aetiology identification, since, even in the course of influenza, its increase does not need to be unequivocally related to a bacterial coinfection—it may also be related to other conditions, such as renal insufficiency [38]. A viral–bacterial differentiation should be treated with caution, since PCT might be related not only to bacterial infection but also to a more severe patient condition in general, a fact that was observed in many patients during the SARS-CoV-2 pandemic [39,40,41]. Nevertheless, procalcitonin adds crucial information to the decision-making process; Esposito et al. showed that PCT-based protocol might decrease the use of antibiotics in pneumonia by approximately 15% [36], not only reducing the number of patients who receive antibiotics but also shortening the duration of antibiotic administration without any harm to the patients. Similarly, Baer et al. proved that a PCT-based protocol might decrease the mean duration of antibacterial treatment in children with LRTIs, especially in children with pneumonia (from 9.1 to 5.7 days) [42]. The study used various PCT cut-offs, including PCT below 0.25 ng/mL (or a 90% decrease in those who had an initial level above 10 ng/mL) [42]. Of note, in our series of patients, the antibiotics were continued much less frequently in children with PCT under 0.25 ng/mL as compared to those with higher values, but the negative predictive value was unexpectedly lower than in naïve children. The above facts highlight the doubts related to the risk of a possible bacterial suprainfection in the course of the influenza and the putative effects of the antibiotics and, consequently, explain the clinicians’ moderation in withdrawal from antibacterial therapy in case of any uncertainty. A PCT-based protocol might facilitate making the decision to stop antibiotic in children with influenza. Wu et al. appreciated PCT’s significant negative likelihood ratio in the meta-analysis on procalcitonin in influenza pneumonia and inferred that, due to the low specificity, PCT may be used mainly as a ruling-out test, especially in patients undergoing intensive care [23].

PCT’s ability to distinguish patients with lower-respiratory-tract involvement was lower (AUC = 0.6) with a moderate NPV (=0.73), yet it is in line with the research by Leng, who included 551 adults with a bloodstream infection and found no differences in PCT concentrations the between upper- and lower-respiratory-tract involvement [37]. Slightly higher AUC and especially NPV (0.63 and 0.83, respectively) was observed for the presence of radiological abnormalities consistent with pneumonia. A recent study on paediatric pneumonia by Florin revealed that PCT seems to reflect the aetiology of an infection rather than the severity of the disease, and in these terms, a PCT-based appraisal of a need for chest X-ray might help diminish the number of unnecessary radiological chest examinations in children with lower procalcitonin values [43].

There are some limitations to this study that need to be addressed. Firstly, this study has a retrospective design, and no PCT-based protocol was used, but in this respect, any prospective experimental trials need to be based on solid foundations from observational studies. Due to the retrospective character, the study itself did not influence the clinical decisions, which were made at the physicians’ discretion only. Secondly, we performed a single-centre study, and multicentre large analyses are necessary, as the group size in our research opens the possibility for more detailed research. Thirdly, we confirmed only one particular bacterial suprainfection, probably mainly due to the low sensitivity of the methods that are currently available; however, a group selection bias cannot be excluded, although we focused on hospitalized patients only. Last but not least, because of practical implications, we deliberately treated influenza as a homogenous disease, which is an obvious simplification. There are (mainly minor) differences not only between the types but also within the subtypes or lineages; however, this was not the focus of the study, since diagnostic methods that can facilitate a determination of the influenza subtypes/lineages are not routinely available to the vast majority of physicians, even in hospital settings.

To conclude, we found procalcitonin to be a useful tool in antibiotic guidance, although, it should be mainly considered as a rule-out, but not a rule-in, test. It should also be emphasized that, in children, procalcitonin may be suggestive of a bacterial suprainfection at lower values than in adults. The implementation of PCT-based protocols might substantially decrease the use of antibiotics in children with influenza.

## Figures and Tables

**Table 1:** Baseline characteristic in the group with and without previous antibacterial therapy (PAT and No-PAT group). Abbreviations: LQ—lower quartile, UQ—upper quartile, CRP—C-reactive protein, PCT—procalcitonin, WBC—white blood cells, ANC—absolute neutrophil count. * Fisher’s exact test.

	No-PAT (*n* = 291)			PAT (*n* = 80)			
	median	LQ	UQ	median	LQ	UQ	*p*
Age (months)	33.0	11.0	64.0	35.5	19.0	61.0	0.117
Duration of signs/symptoms (days)	2.0	1.0	4.0	4.5	2.0	6.0	**0.000**
Duration of fever (days)	2.0	1.0	4.0	4.0	2.0	6.0	**0.000**
Length of stay (days)	5.0	4.0	7.0	6.0	5.0	7.0	**0.013**
Breath rate (per minute)	25.0	22.0	32.0	25.5	20.0	30.0	0.969
Heart rate (per minute)	110.0	100.0	126.0	115.0	100.0	120.0	0.990
CRP (mg/L)	5.42	1.54	14.34	6.72	2.05	20.72	0.325
PCT (ng/mL)	0.18	0.10	0.37	0.15	0.10	0.32	0.337
WBC (*10^3/µL)	7.72	5.35	10.86	7.41	4.77	11.85	0.731
ANC (*10^3/µL)	3.56	2.09	6.32	3.41	2.15	5.47	0.577
Antibiotic frequency (%)	28.9			61.3			0.000 *

**Table 2:** Baseline characteristic in the group without previous antibacterial therapy (No-PAT) according to the need of antibiotic therapy during hospitalization. Abbreviations: LQ—lower quartile, UQ—upper quartile, CRP—C-reactive protein, PCT—procalcitonin, WBC—white blood cells, ANC—absolute neutrophil count.

	Antibiotic Treatment (*n* = 84)			Without Antibiotic (*n* = 207)			
	median	LQ	UQ	median	LQ	UQ	*p*
Age (months)	17.50	8.00	42.5	39.00	13.00	69.00	0.000
Duration of signs/symptoms (days)	3.00	1.00	5.00	2.00	1.00	4.00	0.08
Duration of fever (days)	2.00	1.00	4.00	1.00	1.00	4.00	0.29
Length of stay (days)	7.00	6.00	11.00	5.00	3.00	6.00	0.000
Breath rate (per minute)	29	24	40	24	21	30	0.000
Heart rate (per minute)	120	100	132	108	95	120	0.001
CRP (mg/L)	10.77	3.69	37.75	4.21	1.27	11.13	0.000
PCT (ng/mL)	0.3	0.13	1.15	0.17	0.10	0.29	0.000
WBC (*10^3/µL)	9.27	5.47	14.05	7.26	5.35	9.82	0.003
ANC (*10^3/µL)	4.24	2.05	8.44	3.52	2.09	5.42	0.067

**Table 3:** Procalcitonin performance (at different cut-off values) in the prediction of antibiotic use (No-PAT group), antibiotic continuation (PAT group), lower-respiratory-tract involvement and radiologically confirmed pneumonia. Abbreviations: 95%CI—95% confidence interval, PPV—positive predictive value, NPV—negative predictive value, LRTI—lower-respiratory-tract infection. * Optimal cut-off values according to the Youden index.

Endpoint								
Antibiotic use (No-PAT group)	AUC of 0.658 (95%CI: 0.581 to 0.734, *p* < 0.01)							
Cut-off	Sensitivity	95%CI	Specificity	95%CI	PPV	95%CI	NPV	95%CI
0.4 ng/mL *	46.43%	35.47% to 57.65%	85.51%	79.96% to 90.00%	56.52%	46.49% to 66.04%	79.73%	76.18% to 82.87%
0.25 ng/mL	58.33%	47.06% to 69.00%	68.60%	61.80% to 74.86%	42.98%	36.51% to 49.70%	80.23%	75.61% to 84.15%
0.5 ng/mL	36.90%	26.63% to 48.13%	88.41%	83.24% to 92.43%	56.36%	44.70% to 67.36%	77.54%	74.43% to 80.38%
1 ng/mL	26.19%	17.20% to 36.93%	95.65%	91.91% to 97.99%	70.97%	54.01% to 83.57%	76.15%	73.70% to 78.45%
Antibiotic continuation (PAT group)	0.713 (95%CI: 0.6–0.83, *p* < 0.01)							
Cut-off	Sensitivity	95%CI	Specificity	95%CI	PPV	95%CI	NPV	95%CI
0.23 ng/mL *	54.55%	38.85% to 69.61%	88.89%	70.84% to 97.65%	88.89%	72.69% to 96.01%	54.55%	45.82% to 63.00%
0.25 ng/mL	52.27%	36.69% to 67.54%	88.89%	70.84% to 97.65%	88.46%	71.77% to 95.85%	53.33%	44.94% to 61.55%
0.5 ng/mL	27.27%	14.96% to 42.79%	96.30%	81.03% to 99.91%	92.31%	62.29% to 98.87%	44.83%	40.06% to 49.70%
1 ng/mL	18.18%	8.19% to 32.71%	100.00%	87.23% to 100.00%	100%		42.86%	39.48% to 46.30%
LRTI	AUC = 0.60 (95%CI: 0.53 to 0.66, *p* < 0.01)							
0.27 ng/mL *	49.21%	40.19% to 58.26%	72.24%	66.19% to 77.76%	47.69%	41.07% to 54.40%	73.44%	69.61% to 76.96%
Radiologically confirmed pneumonia	AUC 0.626 (95%CI: 0.56 to 0.7, *p* < 0.01)							
0.27 ng/mL *	55.43%	44.70% to 65.81%	71.68%	66.01% to 76.89%	39.23%	33.20% to 45.61%	82.99%	79.33% to 86.11%

**Table 4:** The results of the multiple regression model for the prediction of the use of antibiotics during hospitalization; the odds ratios of antibiotic implementation in children with lower PCT values in the No-PAT group and the odds ratios of antibiotic continuation in the PAT group are shown. The corresponding percentage of the odds reduction of antibiotic treatment is presented. Abbreviations: No-PAT—no prior antibiotic group, PAT—prior antibiotic therapy group, OR—odds ratio, 95%CI—95% confidence interval.

	Antibiotic Implementation (No-PAT)				Odds Reduction of Antibiotic Treatment		
Cut-off	OR	95%CI		*p*	%	95%CI	
0.25	0.33	0.19	0.57	0.000	67	43	81
0.5	0.22	0.12	0.42	0.000	78	58	88
1	0.12	0.05	0.28	0.000	88	72	95
	Antibiotic continuation (PAT)				Odds reduction of antibiotic treatment		
Cut-off	OR	95%CI		*p*	%	95%CI	
0.25	0.09	0.02	0.44	0.003	91	56	98
0.5	0.12	0.01	0.98	0.048	88	2	99

## Data Availability

Data are available on request from the authors.
